# Individual Differences in Frequency of Inner Speech: Differential Relations with Cognitive and Non-cognitive Factors

**DOI:** 10.3389/fpsyg.2016.01675

**Published:** 2016-11-02

**Authors:** Xuezhu Ren, Tengfei Wang, Christopher Jarrold

**Affiliations:** ^1^School of Education, Huazhong University of Science and TechnologyWuhan, China; ^2^Department of Psychology, Zhejiang UniversityHangzhou, China; ^3^School of Experimental Psychology, University of BristolBristol, UK

**Keywords:** inner speech use, self-talk, individual differences, executive functioning, complex reasoning, impulsivity, trait anxiety

## Abstract

Inner speech plays a crucial role in behavioral regulation and the use of inner speech is very common among adults. However, less is known about individual differences in the frequency of inner speech use and about the underlying processes that may explain why people exhibit individual differences in the frequency of inner speech use. This study was conducted to investigate how individual differences in the frequency of inner speech use are related to cognitive and non-cognitive factors. Four functions of inner speech including self-criticism, self-reinforcement, self-management, and social assessment measured by an adapted version of Brinthaupt's Self-Talk Scale were examined. The cognitive factors that were considered included executive functioning and complex reasoning and the non-cognitive factors consisted of trait anxiety and impulsivity. Data were collected from a large Chinese sample. Results revealed that anxiety and impulsivity were mainly related to the frequency of the affective function of inner speech (self-criticism and self-reinforcement) and executive functions and complex reasoning were mainly related to the frequency of the cognitive, self-regulatory function of inner speech (self-management).

## Introduction

Inner speech, which is usually described as the activity or process of silently talking to oneself (Morin, 2009), has received increasing attention over recent years (e.g., Winsler, 2009; Alderson-Day and Fernyhough, 2015a; Hurlburt and Heavey, 2015; Morin et al., 2015). In the literature, inner speech is also referred to as covert self-talk, verbal thinking, internal dialogue, inner voicing, or self-verbalization. Various theoretical accounts of inner speech and a large body of empirical research have focused on the roles or functions of inner speech in cognitive development and self-regulations (Vygotsky, 1987; Al-Namlah et al., 2006), in working memory capacity (Baddeley et al., 1984, 2001) and also in self-awareness and the acquisition of self-information (Morin, 2005, 2009). There are also a number of studies conducted within the applied and clinical settings where inner speech shows the potential to be an integral component of psychological interventions (e.g., Hardy, 2006; Williams et al., 2012; Shi et al., 2015). Despite these theoretical and empirical advances, relatively less is known about individual differences in the frequency of inner speech use during daily situations, and about the covarying factors that may explain why people exhibit individual differences in the frequency of inner speech use.

One reason why inner speech frequency has not been extensively researched may lies in the common belief that it cannot be easily observed, although there are methods such as the surface electromyography technique that have been developed to detect inner speech production (e.g., Garrity, 1977; Betts et al., 2006; Laurent et al., 2016). Existing research attempting to investigate inner speech frequency mainly relies on self-report methods such as versions of the experience sampling method and questionnaires (cf. Alderson-Day and Fernyhough, 2015b; Morin et al., 2015). For example, using a descriptive experience sampling method that relies on a beeper randomly prompting participants to record their everyday inner experience, Heavey and Hurlburt (2008) found that inner speech, along with other types of inner experience, occurred around 20% of the time as reported by college students. Morin et al. (2011) used an open-format thought-listing procedure to assess the frequency, content, and functions of inner speech. They found that the most reported type of inner speech was self-talk about oneself regarding self-valuations, emotions, physical appearance, and relationships.

Although several questionnaires related to inner speech have been developed (e.g., Brinthaupt et al., 2009, Self-Talk Scale, Calvete et al., 2005; Self-Talk Inventory; Duncan and Cheyne, 1999, Self-Verbalization Questionnaire; McCarthy-Jones and Fernyhough, 2011, Varieties of Inner Speech Questionnaire, VISQ), most do not directly assess the frequency of inner speech use. For example, Duncan and Cheyne's (1999) Self-Verbalization Questionnaire assesses the general tendency to talk to oneself aloud rather than occurrences of covert self-talk. The VISQ assesses four phenomenological properties of inner speech: dialogic quality, condensed quality, evaluative nature, and the extent to which inner speech features the presence of other people. In their initial validation of the VISQ, McCarthy-Jones and Fernyhough (2011) reported that dialogic- and evaluative-inner speech were quite common among people: these two aspects of inner speech were endorsed by 75–80% of participants while the other two properties of inner speech were reported by only a minority. However, the VISQ does not directly measure the frequency of inner speech as it only asks people to rate their levels of agreement with general statements rather than specifically reporting the frequency of inner speech use (see Alderson-Day and Fernyhough, 2015b).

The Self-Talk Scale (STS) developed by Brinthaupt et al. (2009) is an inventory that explicitly measures the frequency of inner speech (along with overt speech) by asking how often a person talks to oneself along four dimensions: self-criticism that encompasses self-blaming talk with negative daily experiences; self-reinforcement that focuses on talking to oneself about positive daily events; self-management pertaining to self-regulation and directing one's daily behavior, and social assessment that refers to one's social interactions. These specific dimensions represent distinct functions of self-talk rather than the contents of what one talks to oneself. In previous validation work, Brinthaupt et al. (2009), Brinthaupt and Dove (2012) and other researchers (e.g., Reichl et al., 2013) reported that individual differences in the STS scores are related to various behavioral and psychopathology traits. In addition, Brinthaupt et al. (2015) recently employed the experience sampling method to examine the accuracy of self-talk frequency measured by the STS. Their results showed that high STS scorers talked to themselves more frequently during recent events compared to low STS scorers. Overall STS scores were also consistent with participants' reports about recent experiences, suggesting that the STS enables accurate measurement of individual differences in the frequency of self-talk. However, the current version of the STS does not exclusively measure the frequency of inner speech use, as it assesses both covert self-talk which is typically called private speech, and overt self-talk that is referred to as inner speech.

Previous studies that have searched for factors associated with the frequency of inner speech use have mostly focused on psychopathological factors such as mood, depression, and anxiety (Calvete et al., 2005; Hatzigeorgiadis et al., 2009; McCarthy-Jones and Fernyhough, 2011; Reichl et al., 2013; Khodayarifard et al., 2014; Shi et al., 2015). For example, Calvete et al. (2005) found that depression and anxiety scores were strongly related to the negative contents of self-talk such as anxious and depressive self-talk. The extent of evaluative inner speech and the presence of other people in inner speech assessed by the VISQ also showed positive correlations with trait anxiety (McCarthy-Jones and Fernyhough, 2011; Alderson-Day and Fernyhough, 2015b). In another study that administered the STS to a sample of university students (Khodayarifard et al., 2014), high frequencies of self-critical and social-assessing self-talk were positively associated with high levels of anxiety and depression. In contrast, high frequency of self-reinforcing self-talk was negatively correlated with depression.

Over and beyond the above factors, there appear to be other important constructs, such as impulsivity, related to individual differences in inner speech frequency. Impulsivity represents a heterogeneous construct that involves a tendency to act on a whim, displaying behavior characterized by little or inadequate forethought (Depue and Collins, 1999). Impulsive people are usually thought to lack attentional resources to control and focus their attention on relevant information and to inhibit distracting information (Whitney et al., 2004). On the other hand, one crucial function of inner speech, described by Hardy (2006) as the cognitive and regulatory function of self-talk, serves to enhance attention focus and to direct and redirect attention (Zinsser et al., 2006). Therefore, impulsive individuals may find themselves disadvantaged when it comes to using inner speech to control their thoughts and behavior. A related finding was reported by Brinthaupt et al. (2009), who showed that frequent self-talkers exhibit high obsessive-compulsive tendencies such as impaired control over mental activities, which suggests that high frequency of inner speech and impulsive thought may be positively associated with each other. However, it is also possible that those who frequently engage in impulsive actions may more often talk to themselves negatively, possibly because of the anxiety, stress or other negative feelings that accompany the impulsive actions (Corr, 2002).

Finally, whether differences in the frequency of inner speech reflect variations of cognitive functioning across people is also an intriguing question. According to Vygotsky's (1987) influential theory, inner speech is crucial for flexible behavioral and cognitive development, and sets the foundation for effective self-regulation. A large body of experimental studies on the cognitive function of inner speech indeed indicates a close relationship between inner speech and cognitive performance (cf. Alderson-Day and Fernyhough, 2015a). For example, blocking inner speech by means of articulatory suppression has been shown to disrupt performance in completing various tasks of working memory and executive functioning (e.g., Phillips et al., 1999; Baddeley et al., 2001; Emerson and Miyake, 2003; Lidstone et al., 2010; Tullett and Inzlicht, 2010; Williams et al., 2012). Although these studies were conducted in experimental settings which may not generalize to daily situations, it can be assumed that those who report higher inner speech frequencies would report higher frequencies of cognitive tendencies. This view was partly tested by Brinthaupt et al. (2009) who collected data on the STS and the Need for Cognition Scale (NCS) which assesses one's tendency to engage in effortful cognitive activities. They found that frequent self-talkers reported higher NCS scores than infrequent self-talkers. However, since the NCS mainly reflects one's self-reported preferences for engaging in cognitive activities, it remains to be seen whether individuals' frequencies of inner speech are directly related to and to what extent reflect their proficiency of cognitive functioning.

The aim of the present study was to investigate how individual differences in the frequency of inner speech use were related to both cognitive and non-cognitive factors including (a) executive functioning, (b) reasoning, (c) trait anxiety, and (d) impulsivity comprising both impulsive thoughts and impulsive actions. According to preliminary evidence and the above assumptions, it was predicted that those cognitive factors including executive functioning and reasoning would be mostly related to the frequency of the cognitive, self-regulatory functions of inner speech, while those non-cognitive factors including anxiety and impulsivity were hypothesized to be mainly associated with the frequency of the affective regulatory functions of inner speech. In particular concerning impulsivity, while impulsive action may be positively related to negative experiences of inner speech, impulsive thinking was expected to be negatively related to the positive experiences of inner speech. Investigating these underlying processes associated with the frequency of inner speech would be important not only for the refinement of intervention programs but also for appropriate applications that take inter-individual differences into account.

Since the STS has been shown to effectively capture the frequency of affective regulatory function (self-criticism and self-reinforcement), the cognitive, self-regulatory function (self-management), and the communicative function (social assessment) of self-talk, this study employed the STS to assess the frequency of inner speech use. However, the STS assesses both private speech and inner speech. Therefore, the instruction of the STS was firstly modified so that participants were aware that only inner speech frequency was being assessed. A battery of cognitive measures was employed to assess executive functioning and reasoning. Anxiety and impulsivity were measured by the State-Trait Anxiety Inventory (Spielberger et al., 1983) and the Barratt Impulsiveness Scale (Patton et al., 1995), respectively. Another novel aspect of the study was that data were collected from a large sample of Chinese university students. Given that the majority of studies of inner speech have been conducted within Western countries, testing these hypotheses concerning inner speech in an Eastern culture has the potential to provide valuable evidence for their external validity.

## Methods

### Participants

A total of 367 undergraduate students from Huazhong University of Science and Technology participated in this study. There were 152 male and 215 female participants aged between 17 and 27 years (*M* = 20.37, *SD* = 1.90). All participants completed the inner speech scales, the anxiety test and the impulsivity test. Forty-five percent of the whole sample, which was 164 students (61 males) with an average age of 20.76 (*SD* = 2.35), additionally completed three executive functioning tasks and two measures of reasoning. In addition, in order to obtain the test-retest reliability of the revised STS, 97 participants (43 males) from the whole sample completed the STS a second time ~3 months after the first testing session. This study was approved by the Human Subjects Review Committee of Huazhong University of Science and Technology. All participants gave written informed consent before entering the study. They were either paid or given course credit for their participation.

### Measures

#### Measure of inner speech frequency

The measure of inner speech frequency was modified from the Self-talk Scale (STS, Brinthaupt et al., 2009). The original STS assesses one's frequency of both covert and overt self-talk. Respondents are asked to indicate how often they talk to themselves by using the common frame “I talk to myself when…” There are 16 items that are all scored in the same direction and rated using a 5-point Likert scale (1, never; 2, seldom; 3, sometimes; 4, often; 5, very often). The scale includes four subscales that assess self-criticism, self-reinforcement, self-management, and social assessment. The self-criticism and self-reinforcement subscales mainly assess the affective regulatory function of self-talk and the self-management subscale mainly reflects the cognitive regulatory function of self-talk. The social assessment subscale mainly reflects the communicative function of inner speech. Table 1 presents the STS's four subscales each including four individual items. Total score is calculated by summing the ratings of the 16 items, with a possible range of 16–80. Sub-scale scores can also be calculated for each type of self-talk with possible ranges of 4–20. Brinthaupt et al. (2009) reported acceptable internal consistencies and good test-retest reliabilities for the scale and subscales.

**Table 1:** **The four subscales of the Self-Talk Scale/the adapted Inner Speech Scale with individual items**.

**Function/types**	**Items [position within the scale]**
Self-criticism	I should have done something differently [1]
	I feel ashamed of something I've done [7]
	I'm really upset with myself [10]
	Something bad has happened to me [14]
Self-reinforcement	Something good has happened to me [2]
	am really happy for myself [5]
	I'm proud of something I've done [8]
	I want to reinforce myself for doing well [13]
Self-management	I need to figure out what I should do or say [3]
	I'm mentally exploring a possible course of action [9]
	I'm giving myself instructions or directions about what I should do or say [12]
	I want to remind myself of what I need to do [15]
Social assessment	I'm imagining how other people respond to things I've said [4]
	I want to analyze something that someone recently said to me [6]
	I try to anticipate what someone will say and how I'll respond to him or her [11]
	I want to replay something that I've said to another person [16]

To meet the aim of assessing only inner speech, we modified the common frame “I talk to myself when…” as shown in the instruction of STS into “I talk to myself *silently* when…” All the other parts of the revised scale were kept the same as the original version. In a next step, the English version of the scale was translated into Chinese. In order to avoid any misunderstanding and to ensure translation accuracy, the Chinese version was blindly back-translated into English, and then translated into Chinese again by two researchers proficient in both Chinese and English. Some slight modifications in phrasing were made so as to make items more appropriate for Chinese respondents. The Results Section presents data on the internal consistency, the test-retest reliabilities for both the total scale and subscales, and the factor structure of adapted version of the inner speech scale (hereafter referred to ISS).

In order to verify that the ISS assesses mainly inner speech, we also collected data using the Varieties of Inner Speech Questionnaire (VISQ, McCarthy-Jones and Fernyhough, 2011) that measures four main phenomenological properties of inner speech including dialogicality, condensed quality, evaluative/motivational nature, and the extent to which inner speech incorporates other people's voices. The VISQ consists of 18 items that measure the four above properties of inner speech. Each item is answered on a 6-point Likert scale from “certainly does not apply to me” (1) to “certainly applies to me” (6). Similarly to the STS, the VISQ was translated and back translated into Chinese.

#### Tests of anxiety and impulsivity

Trait anxiety was assessed by the Chinese version of the Trait scale of the State-Trait Anxiety Inventory (STAI-T, Zhang and Gao, 2012). This scale consists of 20 items that are rated on 4-point rating scale from “Almost Never” (1) to “Almost Always” (4). Half of the items are positively worded (e.g., I am a steady person; I am content) and the other half are negatively worded (e.g., I am nervous and restless; I worry too much over something that really doesn't matter). Scores of positively worded items were reversed in computing the total score so that a greater score on the trait anxiety scale represents greater anxiety. Various applications of this scale to Chinese samples have shown good internal constancy and construct validity (e.g., Yan et al., 2014).

The Chinese version of the Barratt Impulsiveness Scale (BIS-11, Li et al., 2011) was used to assess impulsivity. The scale includes 30 items measuring three broad dimensions of impulsiveness: Motor Impulsiveness (e.g., I do things without thinking), Cognitive Impulsiveness (e.g., I concentrate easily), and Non-Planning Impulsiveness (e.g., I plan tasks carefully). Each dimension consists of 10 items. Each item was rated on a 4-point scale (never/rarely, occasionally, often, and almost always/always). This scale possesses acceptable internal consistency and construct validity (Li et al., 2011).

### Execution functioning tasks

#### Star counting task (SCT)

This task was modified from the original Star Counting Test (De Jong and Das-Smaal, 1995) that was designed to measure the central executive of working memory and has been adapted for computer administration (Ren et al., 2013). Participants had to count the number of stars shown on a series of displays from a starting number in a forward or a backward direction. The presence of a plus or minus sign indicated the direction (forward and backward) in which subsequent stars should be counted. Participants had to continuously update the number of stars in working memory, and to shift between forward and backward counting. At the end of a trial they had to enter the final total into the computer. A detailed introduction of this task was given by Ren et al. (2013). The current task included 24 trials. A correct outcome of counting was coded as one and incorrect outcome as zero for each trial.

#### N-back task

The N-back paradigm has been frequently used to assess working memory updating (Jaeggi et al., 2008; Schmiedek et al., 2009). The current task presented sequences of numbers and asked participants to decide whether each number in a sequence matched the one appearing two items (2-back) or three items (3-back) before. The first two or three numbers were preparatory since they had no references item to be compared with. Participants were asked to press “F” if the numbers were identical or “J” if they were not. This task included both 2-back and 3-back versions each as a block. There were 8 practice trials and 72 test trials. The dependent variable was the number of correct responses.

#### Antisaccade task

This task was modified from Unsworth and Spillers (2010). Each trial started with a fixation point displaying on the screen for a random time from 200 to 2200 ms. A black square was then flashed either to the left or to the right of fixation for 300 ms. Immediately following the square, an arrow (left, right, or up) was presented to the opposite side of the black square for 60 ms, which was followed by a masking stimulus. Participant had to inhibit the prepotent response to the direction of the square in order to respond to the direction of the arrow. There were 10 practice trials and 60 test trials in this task. The number of correct responses to the direction of the arrow was recorded.

### Reasoning scales

#### Raven's advanced progressive matrices

Raven's Advanced Progressive Matrices (APM, Raven et al., 1997). Set II of the APM was adapted for computer administration with a time limit of 30 min. The 36 test items were presented successively by the computer, just as in the paper-and-pencil version. Each item consists of a 3 × 3 matrix composed of geometrical elements, one of which was missing. Participants were instructed to choose an appropriate element out of 8 alternatives by pressing a number key. The responses to each item were recorded as binary data.

#### Horn's reasoning scale

Horn's Reasoning Scale (the 4th scale of LPS, Horn, 1983). This scale consists of 40 individual items. Each item is composed of a series of nine numbers or letters in which eight follow a rule but one does not. The task for the participants was to figure out the rule and to identify the number or letter that did not fit the rule. Participants had to complete the scale in 8 min. The responses to each item were recorded as binary data.

### Procedures

Participants were tested in pairs in a quiet lab. Data on inner speech, anxiety, impulsivity, and the LPS scale were obtained by paper and pencil tests. The other tasks were computer-based measures programmed using E-prime software. There was no time limit for the non-cognitive measures (ISS, VISQ, STAI-T, and BIS). Most Participants spent 10–15 min to complete the non-cognitive measures. The 164 participants who additionally competed the executive functioning tasks and reasoning measures had to take another 60–70 min. The measures were administered in the following order: ISS, VISQ, STAI-T, BIS, SCT, N-back, Antisaccade, APM, and LPS. Participants had an opportunity to take a short break between measures. The re-testing of the ISS was conducted via an online platform. A total of 156 participants were invited to the re-testing session. Ninety-seven of them provided valid re-test data.

## Results

### Descriptive results and reliabilities, and gender differences

Table 2 shows the descriptive statistics, and internal consistency estimates of reliability for each measure (including the subscales of the ISS and VISQ). Data on the cognitive measures were computed based on 164 participants and data on the ISS, STAI-T, and the BIS based on 367 participants. Since three participants' VISQ data were lost, the VISQ scores and subscores were finally computed based on 364 participants. As shown in Table 1, all variables (except the VISQ evaluation subscore) showed normal distributions with values of skewness <2 and kurtosis <4 (see Kline, 2005). Most Cronbach's alphas were in the acceptable range. The relatively low reliabilities for the subscales of the ISS and VISQ were likely due to the limited number of items within each subscale.

**Table 2:** **Descriptive results and reliability estimates (α) of the adapted Inner Speech Scale, the Varieties of Inner Speech Questionnaire, Trait Anxiety Inventory, Barratt Impulsiveness Scale, executive functioning tasks, and reasoning measures**.

**Measure**	**Mean**	***SD***	**Skew**	**Kurtosis**	**α**
1. ISS	56.70	7.91	−0.42	1.65	0.82
2. Self-critical	13.67	2.59	0.01	0.27	0.61
3. Self-reinforcement	13.20	2.85	0.02	−0.06	0.70
4. Self-management	15.61	2.25	−0.73	1.59	0.60
5. Social assessment	14.22	2.97	−0.18	−0.26	0.70
6. VISQ	67.10	11.14	−0.14	0.60	0.76
7. VISQ: dialogic IS	17.39	4.20	−0.71	0.11	0.74
8. VISQ: condensed IS	14.36	5.94	0.64	−0.22	0.81
9. VISQ: other people in IS	14.96	5.33	0.22	−0.34	0.77
10.VISQ: evaluative IS	20.40	2.74	−1.41	4.82	0.68
11. Trait anxiety	44.34	7.69	0.30	−0.48	0.83
12. Motor impulsiveness	15.84	4.37	1.26	1.77	0.80
13. Cognitive impulsiveness	14.89	3.19	0.72	0.20	0.69
14. Non-planning impulsiveness	16.97	4.42	0.56	−0.13	0.76
15. Star counting task	15.49	3.94	−40.40	0.01	0.73
16. Antisaccade task	46.01	11.03	−1.25	0.61	0.93
17. N-back task	45.93	9.18	−0.28	0.08	0.72
18. APM	24.44	5.02	−0.48	0.26	0.83
19. LPS	32.62	3.15	−0.60	1.70	0.71

Data from the cognitive measures were computed based on 164 participants. Data from the VISQ scores were based on 364 participants. ISS, Inner Speech Scale; VISQ, Varieties of Inner Speech Questionnaire; IS, Inner Speech; APM, Raven's Advanced Progressive Matrices, LPS, LPS reasoning scale.

The test-retest reliability for the ISS was also computed. The correlation between total ISS scores at Time 1 and Time 2 reached significance, *r* = 0.66, *p* < 0.001. The correlations between the scores of the two testing sessions for the subscales of the ISS were also significant: self-criticism, *r* = 0.60, *p* < 0.001; self-reinforcement, *r* = 0.66, *p* < 0.001; self-management, *r* = 0.58, *p* < 0.001; and social assessment, *r* = 0.53, *p* < 0.001. These results indicate that the current version of the inner speech scale possesses good near-term stability.

Independent samples *t*-test was used to examine the gender difference in the scores of the ISS. The results showed that there is no significant difference between males and females in either the total score of the ISS (mean diff = −0.23, *t* = −0.28, *p* = 0.78) or each of the subscores of the ISS (Self-criticism: mean diff = 0.06, *t* = 0.23, *p* = 0.82; Self-reinforcement, mean diff = −0.35, *t* = −1.15, *p* = 0.25; Self-management, mean diff = 0.04, *t* = 0.18, *p* = 0.99; and Social assessment: mean diff = 0.01, *t* = 0.02, *p* = 0.78).

### Correlations between variables and the factor structure of the ISS

Table 3 presents the correlation matrix between scores on the ISS, VISQ, and other non-cognitive variables. Table 4 presents the correlation matrix between scores on the ISS and cognitive variables. As shown in Table 2, the correlation between the total scores of the ISS and the VISQ was significant, *r* = 0.49, *p* < 0.001. All subscores of the ISS showed modest to strong correlations with the dialogic, evaluative, and the other people in subscales of the VISQ, indicating that this Chinese version of the inner speech scale reflects these three properties of inner speech measured by the VISQ. The condensed subscale of the VISQ showed virtually no correlation with subscores of the ISS. This condensed subscale showed also no or relatively small correlations with the other three subscales of the VISQ. This correlation pattern was quite similar to those reported by McCarthy-Jones and Fernyhough (2011). Given this, the total score on the VISQ was re-calculated by including only scores of the *dialogic, evaluative*, and *the presence of other people* subscales. This total score showed a relatively strong correlation (*r* = 0.61, *p* < 0.001) with the total score of the ISS.

**Table 3:** **Correlations between scores of the adapted Inner Speech Scale, the Varieties of Inner Speech Questionnaire, the Trait Anxiety Inventory, and the Barratt Impulsiveness Scale (*N* = 367)**.

**Measure**	**1**	**2**	**3**	**4**	**5**	**6**	**7**	**8**	**9**	**10**	**11**	**12**	**13**
1. ISS	–												
2. Self-critical	0.75^**^	–											
3. Self-reinforcement	0.72^**^	0.39^**^	–										
4. Self-management	0.74^**^	0.43^**^	0.40^**^	–									
5. Social assessment	0.76^**^	0.43^**^	0.30^**^	0.46^**^	–								
6. VISQ	0.49^**^	0.35^**^	0.34^**^	0.40^**^	0.37^**^	–							
7. VISQ: dialogic IS	0.49^**^	0.28^**^	0.35^**^	0.43^**^	0.40^**^	0.59^**^	–						
8. VISQ: condensed IS	−0.03	−0.03	0.03	−0.00	−0.09	0.56^**^	−0.10	–					
9. VISQ: other people in IS	0.40^**^	0.33^**^	0.23^**^	0.22^**^	0.39^**^	0.74^**^	0.34^**^	0.13^*^	–				
10. VISQ: evaluative IS	0.53^**^	0.41^**^	0.35^**^	0.53^**^	0.31^**^	0.52^**^	0.40^**^	−0.01	0.28^**^	–			
11. Trait anxiety	0.07	0.29^**^	−0.19^**^	0.01	0.10	0.15^**^	0.03	0.06	0.18^**^	0.08	–		
12. Motor impulsiveness	0.15^**^	0.23^**^	0.04	0.11^*^	0.08	0.19^**^	0.07	0.05	0.21^**^	0.16^**^	0.37^**^	–	
13. Cognitive impulsiveness	−0.18^**^	−0.08	−0.22^**^	−0.17^**^	−0.08	0.02	−0.15^**^	0.15^**^	0.00	−0.02	0.29^**^	0.40^**^	–
14.Non-planning impulsiveness	−0.07	0.08	−0.23^**^	−0.09	0.03	−0.01	−0.06	−0.00	0.05	−0.05	0.34^**^	0.34^**^	0.46^**^

** *Correlations are significant at the 0.01 level*.

* Correlations are significant at the 0.05 level. ISS, the adapted Inner Speech Scale; VISQ, Varieties of Inner Speech Questionnaire; IS, Inner Speech. Correlations involving the VISQ scores were computed based on 364 participants.

**Table 4:** **Correlations between scores of the adapted Inner Speech Scale, executive functioning tasks, and reasoning measures (*N* = 164)**.

**Measure**	**1**	**2**	**3**	**4**	**5**	**6**	**7**	**8**	**9**
1. ISS	–								
2. Self-critical	0.79^**^	–							
3. Self-reinforcement	0.74^**^	0.47^**^	–						
4. Self-management	0.75^**^	0.47^**^	0.40^**^	–					
5. Social assessment	0.79^**^	0.49^**^	0.36^**^	0.52^**^	–				
6. Star counting task	0.07	0.01	−0.01	0.14	0.09	–			
7. Antisaccade task	0.12	0.05	0.02	0.21^**^	0.11	0.35^**^	–		
8. N-back task	0.10	0.06	0.02	0.16^*^	0.09	0.27^**^	0.30^**^	–	
9. APM	0.12	0.04	−0.00	0.22^**^	0.14	0.38^**^	0.37^**^	0.21^**^	–
10. LPS	0.11	0.12	0.09	0.11	0.05	0.32^**^	0.22^**^	0.13	0.44^**^

** *Correlations are significant at the 0.01 level*.

* Correlations are significant at the 0.05 level. ISS, the adapted Inner Speech Scale; APM, Raven's Advanced Progressive Matrices; LPS, LPS reasoning scale.

**Figure 1:**
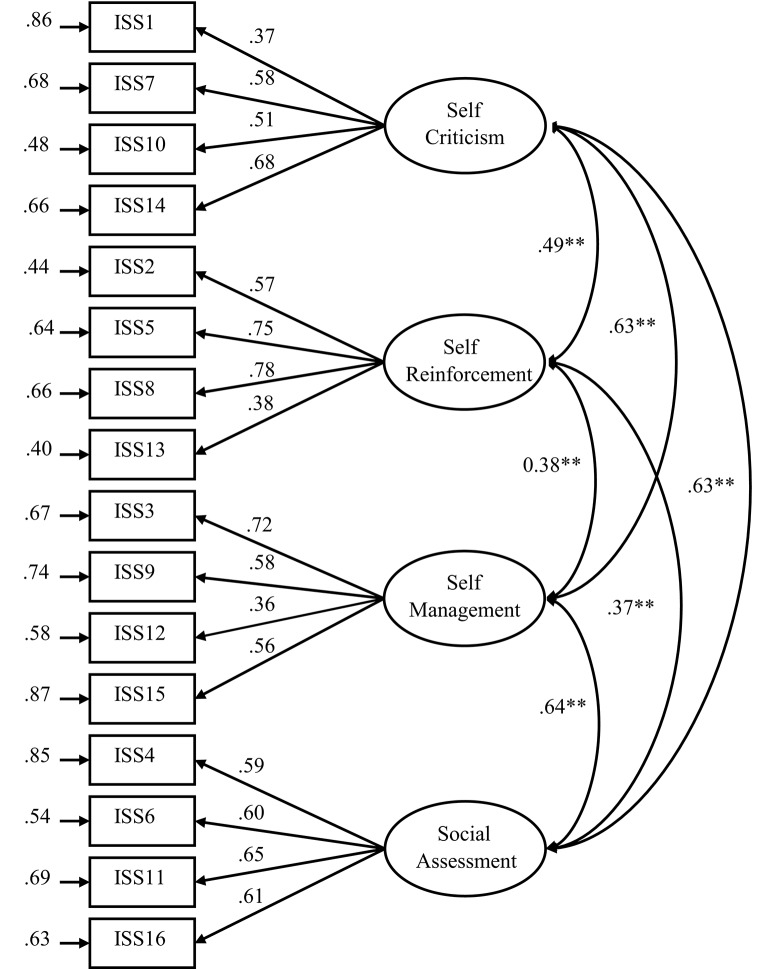
**Illustration of the four-factor structure of the adapted Inner Speech Scale (ISS)**. The four latent variables represent the frequency of four types of inner speech assessed by the ISS. ISS-*n* = the *n*-th item of the ISS (**p < 0.01).

Next, in order to test the factor structure of the ISS, a four-factor CFA model including the four dimensions of inner speech as latent variables and the 16 items of the ISS as manifest variables was estimated. The modeling investigations were conducted by means of LISREL8.8 (Jöreskog and Sörbom, 2006) on the basis of the covariance matrix. As illustrated in Figure 1, this four-factor measurement model showed an acceptable fit to the data, $\chi_{\left(98\right)}^{2}$ = 337.20, RMSEA = 0.08, CFI = 0.90, GFI = 0.90, SRMR = 0.08. Since the four latent factors in Figure 1 showed modest to strong correlations with each other, a model including a second-order factor structure with overall inner speech as the second-order latent variable and self-criticism, self-reinforcement, self-management, and social assessment as the primary factors was estimated. This second-order model showed a similarly acceptable fit to the data, $\chi_{\left(100\right)}^{2}$ = 337.95, RMSEA = 0.08, CFI = 0.90, GFI = 0.90, SRMR = 0.08. These results indicate that the structure of the original STS remained present in the ISS.

### Relationships between frequency of inner speech and non-cognitive variables

**Figure 2:**
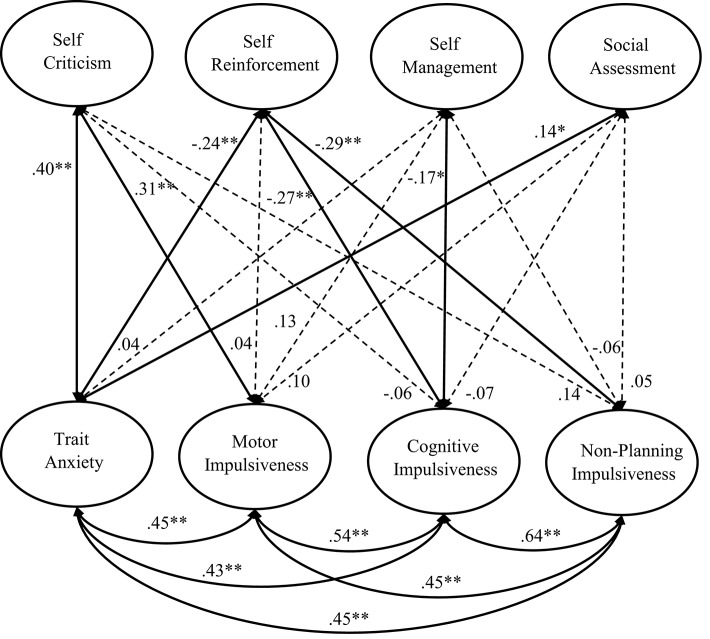
**Illustration of the comprehensive correlation model including the four latent variables representing the four types of inner speech and another four latent variables representing trait anxiety, motor impulsiveness, cognitive impulsiveness, and non-planning impulsiveness**. Self-criticism and self-reinforcement reflect mainly the affective regulatory function of inner speech; self-management reflects mainly the cognitive regulatory function of inner speech; social assessment reflects mainly the communicative function of inner speech. The dotted arrow means that the corresponding correlation was not significant (*p < 0.05, **p < 0.01).

To examine the relationships between the frequency of inner speech and non-cognitive factors, the above four-factor model was extended to a comprehensive correlation model by adding four further latent variables denoted as trait anxiety, motor impulsiveness, cognitive impulsiveness, and non-planning impulsiveness. Five subscores were computed by combining every five neighboring item scores of the anxiety scale, and the subscores served as indicators associated with the Trait Anxiety latent variable. Similarly, each latent variable of impulsivity was represented by two manifest variables computed by summing every five neighboring item scores. This comprehensive model, as illustrated in Figure 2, indicated an acceptable fit, $\chi_{\left(271\right)}^{2}$ = 644.50, RMSEA = 0.06, CFI = 0.93, GFI = 0.88, SRMR = 0.07.

Figure 2 also presents the standardized latent correlations between frequencies of the four types of inner speech and the other latent factors including trait anxiety, motor impulsiveness, cognitive impulsiveness, and non-planning impulsiveness. Trait anxiety was positively correlated with self-criticism but was negatively correlated with self-reinforcement. The correlations between impulsivity and inner speech revealed a more complex picture: while motor impulsiveness was positively correlated with self-criticism, cognitive, and non-planning impulsiveness showed negative correlations with self-reinforcement. In addition, cognitive impulsiveness showed also a small but significant negative correlation with self-management.

### Relationships between frequency of inner speech and cognitive variables

**Figure 3:**
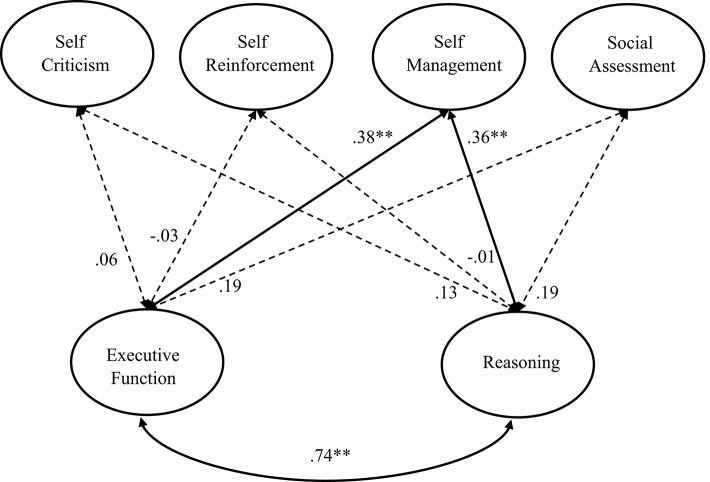
**Illustration of the comprehensive correlation model including the four latent variables representing the four types of inner speech and another two latent variables representing executive functioning and reasoning**. The dotted arrow means that the corresponding correlation was not significant (**p < 0.01).

This section investigates the relationships between the frequency of inner speech and cognitive factors including executive functioning and complex reasoning. We again set up a comprehensive correlation model, in this case by adding two latent variables denoted as executive functioning and reasoning to the four-factor model. The executive functioning latent variable was represented by scores of the SCT, N-back, and Antisaccade tasks. The reasoning latent variable was represented by scores of the APM and LPS reasoning scale. This comprehensive model, as illustrated in Figure 3, also showed an acceptable fit to the data, $\chi_{\left(174\right)}^{2}$ = 277.19, RMSEA = 0.06, CFI = 0.93, GFI = 0.86, SRMR = 0.08. Results regarding the standardized latent correlations between frequencies of the four types of inner speech and the two cognitive variables were clear-cut: only the frequency of self-managing inner speech was significantly and positively correlated with executive functioning and with reasoning. The frequencies of the other three types of inner speech showed no significant correlation with executive functioning or reasoning. In addition, to examine the correlations of the total ISS score with executive functioning and reasoning, the above comprehensive correlation model was reset by including one latent variable representing the total score of the ISS, and the other two latent variables representing executive functioning and reasoning. The fit of this model was close to acceptance, $\chi_{\left(186\right)}^{2}$ = 407.64, RMSEA = 0.09, CFI = 0.86, GFI = 0.80, SRMR = 0.08. The latent variable representing the total score of the ISS showed significant correlations with executive functioning (*r* = 0.21, *p* < 0.05) and reasoning (*r* = 0.22, *p* < 0.05).

## Discussion

The primary purpose of this study was to clarify whether and how frequency of inner speech use relates to both cognitive and non-cognitive factors. Individual differences in four types of inner speech across a variety of everyday situations were examined. The cognitive factors included executive functioning and complex reasoning while the non-cognitive factors consisted of trait anxiety and impulsivity, the latter of which is a multi-faceted construct assessed in the present study by considering three distinct dimensions: motor, cognitive, and non-planning impulsiveness. Structural equation modeling was applied to the data collected from a large number of Chinese university students. Before proceeding to the hypotheses testing, the psychometric properties of the adapted version of the inner speech scale (ISS) were evaluated. This scale exhibited satisfactory internal consistency reliability as well as test-retest reliability. The observation of modest to strong correlations of the ISS with scores of the VISQ (McCarthy-Jones and Fernyhough, 2011) suggests that the ISS did reflect some basic phenomenological characteristics of inner speech. Furthermore, our ISS data based on a Chinese sample supported the four-factor structure of inner speech that was originally reported based on U.S. samples (Brinthaupt et al., 2009), providing cross-cultural evidence for the construct validity of the scale. Structural equation models that linked the frequency of inner speech to cognitive and non-cognitive factors indicated that the frequency of self-critical inner speech had positive relationships with trait anxiety and motor impulsiveness while self-reinforcing inner speech had negative relationships with trait anxiety, cognitive, and non-planning impulsiveness. In contrast, self-management constituted the only function of inner speech that was positively correlated with executive functioning and complex reasoning.

The finding that trait anxiety and impulsivity were mainly correlated with the affective function of inner speech is in line with prior findings that measures of psychopathology are associated with the emotional or affective aspects of self-talk (Calvete et al., 2005). With respect to anxiety, higher levels of anxiety were associated with higher frequency of self-critical but with lower frequency of self-reinforcing inner speech in the current study. These results replicate previous studies conducted using the STS (Brinthaupt et al., 2009, 2015; Khodayarifard et al., 2014) and are also consistent with research within the applied psychology area indicating that self-talk frequency may reflect worries and anxieties in competitive sports (Conroy and Metzler, 2004; Hatzigeorgiadis et al., 2009) or in communication apprehension and public speaking (Shi et al., 2015). The results with respect to impulsivity are in line with previous findings that high frequency of self-talk is associated with obsessive-compulsive tendencies (Brinthaupt et al., 2009), and provide additional evidence that inner speech frequency is associated with various behavioral problems (Alderson-Day and Fernyhough, 2015a). Higher levels of motor impulsiveness were associated with higher frequency of self-critical inner speech, mostly because poorly conceived or unduly risky actions usually result in negative outcomes (Evenden, 1999) which may induce one to think negatively about oneself. On the other hand, higher levels of impulsive thoughts (including cognitive and non-planning impulsiveness) were related to lower frequencies of self-reinforcing and self-managing inner speech, which is in line with the assumption that high impulsive people lack adequate attention resources to use inner speech to reward or regulate their behavior (Hardy, 2006; Zinsser et al., 2006).

The finding that both executive functioning and complex reasoning were significantly related to the frequency of self-managing inner speech is in accordance with theoretical perspectives that highlight the cognitive function of inner speech (Baddeley et al., 1984, 2001; Vygotsky, 1987). It is also in line with existing experimental studies which suggest that inner speech is implicated in various cognitive tasks (Emerson and Miyake, 2003; Lidstone et al., 2010; Tullett and Inzlicht, 2010; Williams et al., 2012). However, the present study differs from previous research in two important ways. First, this study focused on individual variations in the frequency of inner speech and aimed to reveal to what extent variations in inner speech frequency relate to variations in cognitive abilities across people. The revelation of a modest correlation between inner speech and executive functioning or complex reasoning implies that individuals with higher levels of cognitive abilities are better at using inner speech to plan action and to direct behavior. Second, the inner speech investigated in this study is more often used in daily situations compared to those investigated in experimental settings in which inner speech is mostly regarded as a rehearse strategy. Our findings suggest that self-reported methods such as the inner speech scale used in this study are able to capture the cognitive, self-regulatory functions served by inner speech, which has mostly been investigated by means of experimental approaches in the past.

A few limitations of the present study must be mentioned. First, the data were cross-sectional in nature which precludes establishing causal relationships. Therefore, although the results might be taken to imply that individual differences in frequencies of different types of inner speech reflect differences in the associated cognitive and non-cognitive processes measured here, it is also possible that individual differences in those processes are caused by differences in inner speech use. Second, the present study relied on self-reported questions that ask participants to make general judgments about the frequency of inner speech across different situations. There have been concerns over the extent to which data from questionnaires corresponds to ongoing experiences of talking to oneself (e.g., Alderson-Day and Fernyhough, 2015a; Hurlburt and Heavey, 2015). Although recent evidence suggests that scores of the STS correspond well with recent situation-specific inner experiences (Brinthaupt et al., 2015), a combination of questionnaires with other approaches such as experience sampling methods would provide a more accurate assessment of the frequency of inner speech use. Third, the adapted inner speech scale showed only marginal reliabilities, which may be due to the slight change in the instruction or translation issues. Further work is needed to refine this Chinese version of the inner speech scale so as to achieve adequate psychometric qualities.

Despite the above issues, the present study has important implications for research and practice. The results regarding the relation between anxiety and inner speech frequency based on the Chinese sample echo those reported in Western samples (e.g., Calvete et al., 2005; Brinthaupt et al., 2009, 2015; Shi et al., 2015), indicating cross-culture generalizability of the relationship. The result that impulsive action and impulsive thinking related differently to distinct types of inner speech may provide valuable guidance for implementing psychological interventions aimed at diminishing maladaptive behaviors. This study also suggests that self-reported inner speech use across everyday situations to some extent reflect one's cognitive abilities, providing external evidence for experimental research on the cognitive function of inner speech. Furthermore, our study clearly indicates that cognitive abilities are mostly related to the cognitive, self-regulatory function of inner speech while those non-cognitive traits are mainly related to the affective function of inner speech. This constitutes an important empirical step in understanding the underlying processes that may account for individual differences in the frequency of inner speech use.

One extension of the current study would be to alter the instructions of the ISS to reflect private speech rather than inner speech. The frequency of private speech and the associated processes may differ to those of the inner speech. It is particularly important for elucidating individual differences in frequency of both private speech and of inner speech among children given the gradual transition from private speech to inner speech during childhood that was originally proposed by Vygotsky (1987).

## Author contributions

XR contributed to the study design, data analyses, and manuscript drafting. TW contributes to task designs, data analyses, and manuscript development. CJ contributes to the study design and manuscript development.

### Conflict of interest statement

The authors declare that the research was conducted in the absence of any commercial or financial relationships that could be construed as a potential conflict of interest.
